# Genetic Counseling and Germline Testing in the Era of Tumor Sequencing: A Cohort Study

**DOI:** 10.1093/jncics/pkaa018

**Published:** 2020-03-05

**Authors:** Stefan Klek, Brandie Heald, Alex Milinovich, Ying Ni, Jame Abraham, Haider Mahdi, Bassam Estfan, Alok A Khorana, Brian J Bolwell, Petros Grivas, Davendra P S Sohal, Pauline Funchain

**Affiliations:** p1 Loyola University Chicago Stritch School of Medicine, Maywood, IL, USA; p2 Cleveland Clinic Foundation, Taussig Cancer Institute, Cleveland, OH, USA; p3 University of Washington, Seattle Cancer Care Alliance, Fred Hutchinson Cancer Research Center, Seattle, WA, USA; p4 University of Cincinnati Medical Center, Cincinnati, OH, USA

## Abstract

**Background:**

The clinical impact of addressing potential germline alterations from tumor-only next-generation sequencing (NGS) is not well characterized. Current guidelines for cancer genetic testing may miss clinically actionable germline changes, which may have important implications for cancer screening, treatment, and prevention. We examined whether increasing involvement of the clinical genetics service during somatic tumor-only NGS review at Molecular Tumor Board (MTB) increases the detection of germline findings.

**Methods:**

In a retrospective evaluation of patients who underwent tumor-only NGS and were reviewed at MTB, we quantified genetic counseling (GC) referrals as well as germline testing uptake and results across three cohorts: before (C1) and after (C2) the addition of tumor-only NGS review and after (C3) instituting a formal process to coordinate NGS-based genetics referrals to preexisting oncology appointments. All statistical tests were two-sided.

**Results:**

From 2013 to 2017, 907 tumor-only NGS reports were reviewed at MTB (n_C1_ = 281, n_C2_ = 493, n_C3_ = 133); gastrointestinal (22.5%), lung (19.7%), genitourinary (14.8%), and breast (14.1%) were the most common index cancers. GC visits due to MTB increased with each successive cohort (C1 = 1.1%, C2 = 6.9%, C3 = 13.5%; *P* for trend [*P*_trend_] < .001), as did germline testing (C1 = 0.7%, C2 = 3.2%, C3 = 11.3%; *P*_trend_ < .001). Diagnosis of germline pathogenic variants increased with each successive cohort (C1 = 1.4%, C2 = 2.0%, C3 = 7.5%; *P*_trend_ = .003) and with germline pathogenic variants found by MTB review (C1 = 0.4%, C2 = 0.4%, C3 = 2.3%; *P*_trend_ = .12).

**Conclusions:**

Both review of tumor-only NGS by genetics and the institution of a process coordinating GC with oncology appointments increased the discovery of germline pathogenic variants from tumor-only NGS testing. Furthermore, this process identified germline pathogenic variant carriers who would not have otherwise met standard criteria for germline testing.

The clinical impact of addressing potential germline alterations in the era of increasing use of tumor next-generation sequencing (NGS) is not well characterized. Multiple commonly tested genes on tumor-only NGS panels are related to hereditary cancer predisposition (eg, *BRCA1/2*, *TP53*, and *APC*) (1). Currently, most commercial tumor-only NGS panels are offered without paired germline testing and, as such, potential germline alterations found on current tumor-only NGS panels cannot differentiate somatic-only pathogenic variants from those that are germline in origin. Little guidance, and even less data, exist on how to approach suspicious potential germline alterations found on tumor-only NGS. Germline testing and the detection of germline variants have important clinical consequences for both the tested individual and their relatives, because certain germline variants qualify patients for targeted therapies and also have implications for familial disease risk.

Multiple studies have shown a higher-than-expected prevalence of germline pathogenic variants in unselected or minimally selected cancer populations. In a study of 1915 ovarian cancer patients unselected for age of cancer diagnosis or family history, 347 (18%) were found to carry at least one pathogenic germline variant (2). In a study of 450 unselected colon cancer patients diagnosed under the age of 50 years, 72 (16%) were found to have at least 1 cancer-related germline pathogenic variant (3). Of these germline carriers, 33% would not have met clinical germline testing criteria (3). In a second study of unselected colon cancer patients, 14.3% (15 of 105) of germline pathogenic variant carriers lacked clinical history suggestive of an underlying germline pathogenic variant (4). These cohorts suggest that, for a substantial number of individuals, germline pathogenic variants would not be found by interrogation of family history alone; for this subset of patients, population review of tumor-only NGS could be useful in detecting germline pathogenic variants.

Paired tumor-normal sequencing also indicates that a number of individuals who do not meet current clinical germline testing criteria do, in fact, carry germline pathogenic variants, and this type of “incidental” germline finding is not rare. In a single institution cohort that underwent paired tumor-normal sequencing, 17.5% (182 of 1040) were found to have germline pathogenic variants conferring cancer predisposition, of which 101 (55.5%) would not have been referred for germline testing by guideline-directed testing (5). These data suggest that universal sequencing of paired tumor-normal DNA may detect additional germline variants that an approach based on current clinical guidelines alone would not.

Currently, the most commonly ordered tumor-only NGS panels involve tumor-only sequencing and do not include germline testing. We hypothesized that reviewing tumor-only test results for potential germline alterations, via inclusion of cancer genetic counselors (GC) at Molecular Tumor Board (MTB) and enacting a clinical workflow that integrated genetics appointments within existing cancer clinic appointments, would increase the rate of germline pathogenic variant detection.

## Methods

### Study Population and Procedures

All adult patients with a solid tumor malignancy who underwent tumor-only NGS and MTB review at Cleveland Clinic between September 2013 and November 2017 were reviewed. Genomic sequencing of 315 cancer-related genes was performed using the FoundationOne assay (Cambridge, MA). Chart review assessed the timing of GC visits in relation to tumor-only NGS testing and MTB review, whether GC visits were due to MTB recommendations, what percentage of patients completed GC visits and underwent germline testing, and germline testing results.

Three cohorts—C1, C2, and C3—were analyzed. Cohort C1 designates the period from September 2013 to January 2015, during which tumor-only NGS results underwent MTB review without clinical genetics input. Cohort C2 designates the period from January 2015 to July 2017, during which a cancer geneticist reviewed tumor-only NGS results for potential germline alterations. During this period, MTB notes reflected recommendations for potential germline alterations when appropriate. Referrals to genetics required the primary oncologist to place a genetics referral and patients to schedule with genetics. Cohort C3 describes the period from August 2017 to November 2017, during which an opt-out system for automatic genetics referrals from MTB was operationalized. After email notification of potential germline alterations from tumor-only NGS reports, primary oncologists would have a designated time period in which to communicate a patient or provider preference to opt out of a GC visit. If that time elapsed without an opt-out request, a GC visit would be coordinated with a provider visit or treatment time to discuss the identified potential germline alterations and decide on whether germline testing would be pursued.

Tumor-only NGS results were reviewed for potential germline alterations following the American College of Medical Genetics and Genomics medically actionable gene list (6) plus about 40 other clinically relevant genes associated with cancer predisposition. In addition, the type and location of the genetic alteration, variant allele fraction, tumor type, family history, and age of cancer diagnosis were taken into consideration (Box 1). Although multiple publications suggest that guidelines do not capture all germline pathogenic variant carrier status (2–4), tumor type and family history were taken into account, especially in cases of selected genes (eg, *APC*, *NF*, and *TSC*), where one would expect to find physical features (eg, neurofibromas in *NF1*) or evidence of nonmalignant pathology (eg, polyposis in *APC*). In such cases, genetic counselors used clinical judgment in the absence of these features to recommend against genetic testing.

**Box 1.** Rules applied to tumor-only NGS review to identify potential germline alterations

If somatic variant found in a gene on potential germline list*	
a.	Include *BRCA1/2*, any variant
b.	Exclude amplification
c.	Exclude *APC* unless double mutants, I1307K, or polyposis
d.	Exclude *TP53* unless double mutant
e.	Exclude *CDKN2A* unless double mutant
f.	Exclude *TERT* unless c.-57 T>G variant
g.	Exclude *POLE* and *POLD1* if not exonuclease domain (codons 268–471 of *POLE* and codons 304–517 of *POLD1*)
h.	Exclude *PTCH1* unless basal cell carcinoma
i.	Exclude *TSC1/2* unless clinical phenotype suggestive of tuberous sclerosis complex
j.	Variant allele frequency ≥35%

*Potential germline gene list: *APC, ATM, BARD1, BLM, BMPR1, BRCA1, BRCA2, BRIP1, CDH1, CDKN2A, CEBPA, CHEK2, EPCAM, FANCC, FH, FLCN, GATA2, HOXB13, KIT, MAX, MEN1, MET, MITF, MLH1, MSH2, MSH6, MUTYH, NBN, NF1, NF2, PALB2, PDGFRA, PMS2, PTCH1, PTEN, RAD50, RAD51, RB1, RET, RUNX1, SDHA, SDHAF2, SDHB, SDHC, SDHD, SMAD4, STK11, TERC, TERT, TET2, TP53, TSC1, TSC2, VHL, WT1, and XRCC2.*

The following genes were considered on a case-by-case basis (rare): *ALK, AXIN2, BAP1, CASR, CDC73, CDK4, CDKN1B, CDKN1C, DIS3L2, EGFR, ERBB2, ETV6, GPC3, GREM1, HRAS, MC1R, MRE11A, PARK2, PHOX2B, POLD1, POLE, PRKAR1A, RECQL4, SMARCA4, SMARCB1, SMARCE1, SUFU, and WRN*.

The study was approved by the Cleveland Clinic Institutional Review Board.

### Statistical Analysis

Study data were collected and managed using Research Electronic Data Capture tools hosted at Cleveland Clinic. Descriptive results are provided using median (range) and percentage values. *P* for trend (*P*_trend_) was calculated by Extended Mantel-Haenszel χ^2^ for linear trend. All tests were two-sided and *P* value of less than .05 was considered statistically significant.

## Results

### Patient Characteristics

Clinical characteristics are provided in Table 1. A total of 907 patients had tumor-only NGS reports reviewed at MTB from 2013 to 2017 (n_C1_ = 281, n_C2_ = 493, n_C3_ = 133). Median age at the time of tumor-only NGS testing was 61.9 years (range, 19.5–93.8 years), and the median time to tumor-only NGS testing from time of initial cancer diagnosis was 1.6 years (range, 0.0–16.0 years). Overall, 86.8% of patients were white and 50.8% were female, with gastrointestinal (22.5%), lung (19.7%), genitourinary (14.8%), and breast (14.1%) as the most common index cancer diagnoses.

**Table 1.** Patient characteristics of the study population

	C1 (n = 281)	C2 (n = 493)	C3 (n = 133)	Total (n = 907)
Variables	No. (%)	No. (%)	No. (%)	No. (%)*
Sex				
Female	145 (51.6)	247 (50.1)	69 (51.9)	461 (50.8)
Male	136 (48.4)	246 (49.9)	64 (48.1)	446 (49.2)
Race				
White	247 (87.9)	426 (86.4)	114 (85.7)	787 (86.8)
Black	22 (7.8)	42 (8.5)	13 (9.8)	77 (8.5)
Other	12 (4.3)	25 (5.1)	6 (4.5)	43 (4.7)
Age at time of tNGS^†^, y				
Median age at tNGS (range)	59.3 (24.2–93.8)	63.4 (19.5–92.0)	60.9 (22.9–85.9)	61.9 (19.5–93.8)
Median time of tNGS from initial dx (range)	1.5 (0.0–13.3)	1.8 (0.0–14.7)	1.6 (0.1–16.0)	1.6 (0.0–16.0)
Cancer diagnosis				
Gastrointestinal	78 (27.8)	98 (19.9)	28 (21.1)	204 (22.5)
Lung	60 (21.4)	104 (21.1)	15 (11.3)	179 (19.7)
Genitourinary	19 (6.8)	80 (16.2)	35 (26.3)	134 (14.8)
Breast	60 (21.4)	55 (11.2)	13 (9.8)	128 (14.1)
Head and neck	43 (15.3)	44 (8.9)	11 (8.3)	98 (10.8)
Gynecologic	2 (0.7)	31 (6.3)	16 (12)	49 (5.4)
CUP	11 (3.9)	31 (6.3)	3 (2.3)	45 (5)
Melanoma	4 (1.4)	33 (6.7)	4 (3)	41 (4.5)
Other^‡^	4 (1.4)	17 (3.4)	8 (6)	29 (3.2)

* Because of rounding, percentages may not total 100. dx = diagnosis; C1 = cohort of MTB cases occurring without clinical genetics review; C2 = cohort of MTB cases after the addition of clinical genetics review; C3 = cohort of MTB cases after instituting a formal process to coordinate tNGS-based genetics referrals to preexisting oncology appointments; CUP = cancer of unknown primary; tNGS = tumor-only next-generation sequencing.

^†^ tNGS.

^‡^ Other includes cancers of the bone, connective tissue, thyroid, and lymphoma.

### Overall GC and Germline Testing Uptake

Within this 907-patient cohort, 182 (20.1%) had at least 1 GC visit. Of 182 patients who underwent GC, 44 (24.2%) did not pursue germline testing; reasons included that genetic testing was not clinically indicated after pedigree and chart review by GC (n = 28), patients lost to follow-up (n = 10), and insurance denial of testing (n = 6). Of the 138 who underwent germline testing, 114 (82.6%) were found not to harbor a germline pathogenic variant; 24 (17.4%) were found to carry a germline pathogenic variant conferring cancer predisposition (Table 2).

**Table 2.** Referrals, visits, and germline testing results*

	C1 (n = 281)	C2 (n = 493)	C3 (n = 133)	Total (n = 907)	
Variables	No. (%)	No. (%)	No. (%)	No. (%)	*P*_trend_ ^†^
GC visits (% of tNGS)	41 (14.6)	97 (19.7)	44 (33.1)	182 (20.1)	<.001
Due to MTB recommendations or workflow	3 (1.1)	34 (6.9)	18 (13.5)	55 (6.1)	<.001
Independent of MTB	38 (13.5)	63 (12.8)	26 (19.5)	127 (14.0)	.23
Germline testing (% of tNGS)	31 (11.0)	69 (14.0)	38 (28.6)	138 (15.2)	<.001
Due to MTB recommendations or workflow	2 (0.7)	16 (3.2)	15 (11.3)	33 (3.6)	<.001
Independent of MTB	29 (10.3)	53 (10.8)	23 (17.3)	105 (11.6)	.09
(+) GL findings (% of tNGS)	4 (1.4)	10 (2.0)	10 (7.5)	24 (2.6)	.003
Due to MTB recommendations or workflow	1 (0.4)	2 (0.4)	3 (2.3)	6 (0.7)	.12
Independent of MTB	3 (1.1)	8 (1.6)	7 (5.3)	18 (2.0)	.02

* Patients whose tNGS reports were presented at MTB before (C1) and after (C2) the addition of review of tNGS for potential germline alterations by clinical genetics at MTB and after (C3) the implementation of a formal integrated cancer genetics approach to coordinate post-MTB GC and integrate patients’ genetics appointments within their existing cancer appointments. GC = genetic counseling; MTB = Molecular Tumor Board; tNGS = tumor-only next-generation sequencing.

† All *P* values calculated by Extended Mantel-Haenszel χ^2^ for linear trend.

### Comparison of Cohorts With Differing Involvement of Clinical Genetics During Tumor-Only NGS Review

The overall study cohort was subdivided temporally into 3 subcohorts based on the level of GC involvement in MTB: C1, with no formal review of tumor-only NGS for potential germline alterations (during which GC referrals occurred solely due to preexisting practice patterns for medical genetics referrals, eg, clinicians identifying “red flags” in patients’ personal or family histories); C2, during which a cancer geneticist reviewed all tumor-only NGS results and formal recommendations for GC were made within MTB based on both American College of Medical Genetics and Genomics-determined actionable genes and other clinically relevant genes associated with cancer predisposition (Box 1); and C3, after institution of an clinical process streamlining the coordination of MTB-recommended GC consults with preexisting oncology appointments.

Within each cohort, the proportion of GC visits, germline testing, and germline pathogenic variant findings increased with each successive cohort. GC visits increased from C1 to C2 to C3 (14.6% to 19.7% to 33.1%, *P*_trend_ < .001); germline testing increased from C1 to C2 to C3 (11.0% to 14.0% to 28.6%, *P*_trend_ < .001); and, similarly, germline pathogenic variant findings increased from C1 to C2 to C3 (1.4% to 2.0% to 7.5%, *P*_trend_ = .003) (Table 2).

Furthermore, when these trends were evaluated specifically for those cases where MTB directly influenced germline follow-up, we found that GC visits increased from C1 to C2 to C3 (1.1% to 6.9% to 13.5%, *P*_trend_ < .001); germline testing increased from C1 to C2 to C3 (0.7% to 3.2% to 11.3%, *P*_trend_ < .001); and the discovery of germline pathogenic variants increased in C3, without the trend reaching statistical significance (C1 to C2 to C3, 0.4% to 0.4% to 2.3%, *P*_trend_ = .12). (Table 2).

### Characterization of Pathogenic Variant Carriers

Of 24 patients who were found to carry a germline pathogenic variant, 15 (62.5%) were female and 21 (87.5%) were white. Common index cancers were breast (29.2%), gastrointestinal (20.8%), prostate (16.7%), and gynecologic (16.7%) (Table 3). Germline pathogenic variants conferring cancer predisposition were found in the following 12 genes: *ATM*, *BAP1*, *BRCA1*, *BRCA2*, *CDH1*, *MLH1*, *MSH2*, *MUTYH*, *PTEN*, *SDHB*, *SDHA*, and *TP53* (Table 4); 7 patients had pathogenic variants in *BRCA2* and 4 patients in *BRCA1* (Table 4). One-fourth (n = 6; 3 men) of patients with germline pathogenic variants in this cohort were diagnosed directly due to an MTB recommendation (Table 4), including 3 with prostate cancer, 2 with breast cancer, and 1 with melanoma. Of note, all 24 germline pathogenic variants found on germline testing were concordant with the corresponding tumor-only NGS (data not shown).

**Table 3.** Demographic and clinical characteristics of germline pathogenic variant carriers

	Germline pathogenic carriers (n = 24)
Variables	No. (%)*
Sex	
Female	15 (62.5)
Male	9 (37.5)
Race	
Black	1 (4.2)
Other	2 (8.3)
White	21 (87.5)
Age at time of tNGS^†^, y	
Median age at tNGS	61.9
(range)	(23.8–74.0)
Median time of tNGS from initial dx	4.2
(range)	(0.1–16.0)
0–44	4 (16.7)
45–64	13 (54.2)
65–74	7 (29.2)
≥75	0 (0)
Cancer dx	
Breast	7 (29.2)
Gastrointestinal	5 (20.8)
Prostate	4 (16.7)
Gynecologic	4 (16.7)
Lung	2 (8.3)
Brain	1 (4.2)
Melanoma	1 (4.2)

* Because of rounding, percentages may not total 100. dx = diagnosis; tNGS = tumor-only next-generation sequencing.

† tNGS.

**Table 4.** Genetic testing details of germline pathogenic variant carriers, findings due to MTB, and concordance with tNGS*

ID	Sex	Cancer diagnosis	Due to MTB	Germline panel findings	Concordance with tNGS	Pathogenic variant
1	M	Prostate	No	*ATM*	Yes	V1268fs*1
2	F	Ovarian	No	*ATM*	Yes	H1082fs*14
3	M	Melanoma	Yes	*BAP1*	Yes	G594fs*48
4	F	Breast	No	*BRCA1*	Yes	E23fs*17
5	F	Breast	No	*BRCA1*	Yes	Q1756fs*74
6	F	Breast	No	*BRCA1*	Yes	K894fs*8
7	F	Ovarian	No	*BRCA1*	Yes	K894fs*8
8	F	Lung	No	*BRCA2*	Yes	L2357fs*2
9	F	Colorectal	No	*BRCA2*	Yes	N1544fs*4
10	M	Prostate	Yes	*BRCA2*	Yes	K437fs*22
11	F	Endometrial	No	*BRCA2*	Yes	Q969*
12	F	Peritoneal	No	*BRCA2*	Yes	S1982fs*22
13	M	Prostate	Yes	*BRCA2*	Yes	Y1710fs*1
14	M	Prostate	Yes	*BRCA2*	Yes	E1035*
15	M	Gastric	No	*CDH1*	Yes	W526*
16	M	Colon	No	*MLH1*	Yes	Q537fs*54
17	M	Prostate	No	*MSH2*	Yes	loss exons 1–6
18	F	Breast	Yes	*MUTYH*	Yes	Y165C
19	F	Breast	No	*PTEN*	Yes	Y16fs*28
20	F	Head and neck	No	*SDHB*	Yes	F238fs*10
21	M	Colon	No	*SDHA*	VUS^†^	A454E
22	F	Lung	No	*SDHA*	Yes	R31*
23	F	Breast	Yes	*TP53*	Yes	R158P
24	F	Breast	No	*TP53*	Yes	Y220C

* Table organized alphabetically by germline pathogenic variant. All 24 germline pathogenic variants found on germline testing were concordant with the corresponding tNGS, with 23 of the 24 variants appearing on the front page of the report and 1 of the 24 appearing in the VUS section of the report. MTB = Molecular Tumor Board; tNGS = tumor-only next-generation sequencing; VUS = variants of unknown statistical significance.

† This finding appeared in the variants of unknown significance section of the report.

Each of the 24 verified germline pathogenic variants had its corresponding somatic variant reported within tumor-only NGS results; the somatic variant was reported in the main body of the report for 23 of the 24 and appeared within the variants of unknown statistical significance section for 1 of the 24 (Table 4).

## Discussion

This study characterizes the clinical impact of reviewing tumor-only NGS for potentially germline findings in the context of an MTB and with the subsequent addition of an integrated, formal clinical genetics referral process. The American Society of Clinical Oncology recommends that providers communicate the potential for incidental or secondary germline findings before tumor-only NGS is ordered to review potential benefits and limitations (7). Providers should determine patient preferences regarding the receipt of germline information and allow patients to decline, and providers should communicate germline findings from tumor-only NGS in clinical settings (7). In this effort, we aimed to quantify the clinical impact of a directed effort to evaluate tumor-only NGS results for germline implications and streamline resulting referrals to GC.

Our data suggest that a conscious effort to address tumor-only NGS results for germline implications yields an increase in both the proportion of patients completing a genetics consultation and the proportion of patients undergoing germline testing. Steady increases from C1, a cohort with no genetics input at MTB; to C2, the addition of a cancer geneticist to MTB review; to C3, initiation of a workflow that coordinated GC visits, indicate that each clinical step was additive to the observed increases in GC referrals and, ultimately, germline testing. At an academic center with a well-established cancer GC department, and thus a relatively high GC uptake at baseline, the additive contributions of MTB review as well as an integrated clinical referral process to the uptick in GC visits is even more notable. This increase in GC visits due to MTB suggests the existence of patient subsets that were previously not referred or did not complete referrals; our data suggest that these subsets can benefit from implementation of MTB-centered referral processes.

Genetic testing also rose with each successive cohort, and this statistically significant increase was specific to the MTB-referred cohorts. Observation of a parallel increase in both germline testing and GC visits suggests that the additional MTB-driven referrals were appropriate and specific; in other words, patients sent to GC by MTB warranted subsequent testing when evaluated within a GC visit. Nearly one-third of the C3 cohort underwent GC, and the vast majority who saw GC had germline testing (38 of 44, 86%). In the C1 cohort, a lower percentage of GC visits resulted in germline testing (31 of 41, 76%). Although not reaching statistical significance, these numbers imply that MTB-driven referrals result in germline testing in a proportion at least equivalent to, if not higher than, prior MTB-independent referral practices.

Germline pathogenic variant detection did statistically significantly increase over the study period, from 1.4% to 7.5%. This increase was not observed to be specific to MTB referrals, although a trend would be difficult to ascertain given that the absolute numbers of germline pathogenic variants were relatively small. Carriers of germline pathogenic variants comprised 1.4% of the C1 cohort, comparable with a previously cited study of paired tumor-normal NGS. In that study, 1.8% (182 of 10 336) of patients were found to have pathogenic germline variants conferring cancer predisposition. Similar to the C1 cohort in our study, patients in that study were first referred to clinical genetics by their oncologists, presumably due to standard-of-care guidelines (5). After adding germline MTB review and instituting a streamlined clinical genetics referral process in our population, the observed increase from 1.4% to 7.5% of identified germline pathogenic variant carriers suggests that the true number in advanced cancer is much higher than the percentage traditionally uncovered by standard-of-care referral practices. An automated, single institution experience in addressing germline review for somatic testing found a 5% referral rate with 74% germline confirmation (8). A second single institution using paired tumor-normal sequencing referred 6.4%, with 63% true germline yield (9). The lower rate in these studies is likely due to stricter criteria from automated review and smaller gene lists of interest. Addressing germline implications of somatic testing within oncologic practices could contribute to identifying greater numbers of patients with cancer who carry a hereditary cancer predisposition.

Although tumor-only NGS would certainly not be appropriate as a solitary germline screening tool, in a subset of pathogenic variant-positive patients, germline pathogenic variants were discovered solely due to MTB. Of 24 germline pathogenic variants diagnosed, 6 (25.0%) were found as a direct result of MTB recommendations. Four (66.7%) would not have met standard clinical guidelines for genetic testing. Given the ability to identify germline pathogenic variants in patients who otherwise would not have met guideline-based germline testing criteria, tumor-only NGS review represents a method that may be complementary, but not interchangeable, to current germline screening approaches.

Concordance of commercial germline sequencing with corresponding tumor-only NGS results was high, with all 24 verified germline pathogenic variants having a corresponding somatic variant reported within tumor-only NGS. Such high concordance suggests that a large number of germline pathogenic variants would be identified using tumor-only NGS, indicating that tumor-only NGS may be used as an additional, but not adequate, mechanism to screen patients for germline testing. Most tumor-only NGS panels do not provide complete coverage of all genes with potential germline implications and thus cannot detect all germline pathogenic variants. Certain pathogenic germline variants are difficult to identify even with dedicated germline sequencing including large rearrangements, those that occur within genes that share large segments with pseudogenes, promoter variants that lie outside of exons, and those related to germline methylation. Such variants represent easily missed germline diagnoses in tumor-only NGS testing, which is not inherently designed to detect, or report, germline findings. Thus, tumor-only NGS should not be used exclusively as a screening tool for genetic risk assessment and cannot replace critically important dedicated germline tests.

Other practical considerations regarding MTB review for germline findings include patient consent, particularly with an opt-out referral process, and duty to warn in the posthumous setting. In our study, only 1 patient refused GC in the automatic GC referral (C3) cohort. Similarly, in a study of 1167 patients who underwent paired tumor-normal sequencing, 1157 (99.1%) desired to be informed of incidental germline findings (10). Additionally, some in our study declined germline testing after GC or were unable to procure insurance coverage for testing. Others were lost to follow-up or death. Unlike pathogenic variants found by commercial germline sequencing, where posthumous contact of family members is justified by the concept of duty to warn, it is unclear the same duty to warn applies to potential germline alterations found through tumor-only NGS. In our practice, if a provider has a reason to suspect that a somatic variant is germline and the patient is deceased, it can be justified to contact the patient’s relatives to relay the potential for cancer risk and provide an avenue to pursue further genetic consultation and testing.

Limitations of our study include its single center, retrospective nature and a predominantly white population, which may limit the generalizability of our findings. Additional limitations include potential patient selection and referral biases affecting which patients were referred for tumor-only NGS and GC. Important to note, this study was conducted during a period of explosive growth in somatic testing. Whether increases in somatic testing heightened an awareness of genetics and led to more GC referrals independent of MTB could not be ascertained. However, no statistically significant upward trend was observed with GC referrals that occurred independent of MTB, suggesting that many of the overall upward trends were driven by MTB recommendations.

To our knowledge, this study marks the first expansive attempt to quantify the clinical outcomes resulting from formally assessing for potential germline alterations from tumor-only NGS and implementing a clinical process to handle clinical recommendations from this type of assessment. In patients with advanced cancer who undergo tumor-only NGS, such a process may help lead to the discovery of pathogenic germline alterations that would have otherwise gone undetected using only current guidelines for cancer genetic testing. Current guidelines for genetic risk assessment referral may miss clinically actionable germline changes. An increasing number of patients undergo tumor-only NGS to seek out additional therapeutic options. These results are not currently used to their fullest potential. Our data suggest thorough review by clinical genetics for potential germline alterations, ideally in a context similar to MTB and with a clinical process to enact MTB recommendations, is likely to increase identification of individuals carrying a heritable cancer predisposition and add value to somatic NGS testing. Although the review of tumor-only NGS results for potential germline alterations should not substitute for detailed family history, age of cancer diagnosis, and other commonly used clinical criteria, it can be a complementary way to increase GC use and detection rate of germline pathogenic variants in patients and their families.

## Funding

None applicable.

## Notes

Stefan Klek, Alex Milinovich, Ying Ni, Jamie Abraham, Bassam Estfan, Brian James Bolwell, and Pauline Funchain have nothing to disclose.

Brandie Heald: consulting or advisory role: Invitae; and speakers' bureMyriad Genetics. Haider Mahdi: research funding: Puma Biotechnology. Alok A. Khorana: honoraria: Parexel, Pfizer, Sanofi, Janssen, Halozyme, Seattle Genetics, AngioDynamics, Leo Pharma, Medscape/WebMD, Pharmacyclics, PharmaCyte, TriSalus, and Bayer; consulting or advisory role: Pfizer, Janssen, Bayer, and Sanofi; research funding: Janssen, Merck (Inst), Array (Inst), Bristol-Myers Squibb (Inst), and Leap (Inst); and travel, accommodations, expenses: Parexel, Pfizer, Sanofi, Halozyme, Seattle Genetics, AngioDynamics, Leo Pharma, Medscape/Web MD, Bayer, and Janssen. Petros Grivas: consulting or advisory role: Merck, Bristol-Myers Squibb, AstraZeneca, Clovis Oncology, EMD Serono, Seattle Genetics, Foundation Medicine, Driver, Inc, Pfizer, QED Therapeutics, HERON, Janssen, Bayer, Genzyme, Mirati Therapeutics, Exelixis, Roche, GlaxoSmithKline; and research funding: Pfizer (Inst), Clovis Oncology, Bavarian Nordic (Inst), Immunomedics (Inst), Bristol-Myers Squibb (Inst), and Debiopharm Group (Inst). Davendra Sohal: honoraria: Foundation Medicine; consulting or advisory role: Perthera; research funding: Bayer (Inst), Celgene (Inst), Genentech (Inst), Novartis (Inst), and OncoMed (Inst); and travel, accommodations, expenses: Foundation Medicine.
